# Supplementary material for: E3 Ubiquitin Ligase PUB23 in Kiwifruit Interacts with Trihelix Transcription Factor GT1 and Negatively Regulates Immune Responses against Pseudomonas syringae pv. actinidiae
Source: Int J Mol Sci. 2024 Feb 5;25(3):1930. doi: 10.3390/ijms25031930 (PMC10856358; doi:10.3390/ijms25031930)
Supplement: Supplementary file 1 [file ijms-25-01930-s001.zip › ijms-2849346-supplementary.pdf]

Table S1. list of primers used in this article

Gene name	Forward primer (5'->3')	Reverse primer (5'->3')
Actin	TGCATGAGCGATCAAGTTTCAAG	TGTCCCATGTCTGGTTGATGACT
PR1	GCCCCCGGTAAGGTTTGT	CGAACCAAGACCCACTATTGC
RIN4	CTTTGAGAAAGCACGGAAGG	TGTTCTTCTGGTTCGGTTC
GT1	AACGCCCAAACCAAACCCTA	AAACCTCAGAATCGCCCTCG
ICS1	AGGCGAGGCTTCTAATTG	ACAGCAAACTCACTCTCTC
